# The impact of Fc glycosylation on IgG susceptibility to hinge region chemical reduction: implications for the development of immunoassays

**DOI:** 10.1016/j.bbrep.2025.102112

**Published:** 2025-06-25

**Authors:** Vanessa Susini, Silvia Ursino, Chiara Sanguinetti, Alice Botti, Laura Caponi, Maria Franzini

**Affiliations:** Department of Translational Research and of New Surgical and Medical Technologies, University of Pisa, Via Savi 10, Pisa, Italy

**Keywords:** IgG glycosylation, Chemical reduction of the hinge region, Immunoassay

## Abstract

Antibodies are glycoproteins, and Fc glycosylation plays a critical structural role in maintaining the proper folding of the C_H_2 domain. Deglycosylated IgGs exhibit an open C_H_2 domain conformation that structurally affects also the neighboring hinge region. Selective reduction of disulfide bonds in this region using mild reducing agents generates monovalent thiol-containing IgGs (rIgGs), which can be immobilized on modified surfaces to orient the Fab fragment outward, enhancing antigen binding efficiency and assay sensitivity. This study investigates the effect of Fc glycosylation on IgG chemical reduction and its implications for in-house ELISA and commercial immunoassays. IgGs were enzymatically deglycosylated with Endo S and then reduced to rIgGs by 2-mercaptoethylamine. Deglycosylation and reduction efficiency were verified by non-reducing SDS-PAGE. Glycosylated and deglycosylated rIgGs were tested in in-house ELISA and commercial immunoassays. Results showed that deglycosylation significantly improves rIgG production, probably by increasing hinge-region accessibility to reducing agents. This led to a 20-fold increase in ELISA sensitivity compared to glycosylated rIgGs. Deglycosylation also mitigates batch-to-batch variability in IgG reduction, enabling consistent rIgG yields. These findings highlight the capability of deglycosylation to standardize rIgG production, broadening its applications in diagnostic immunoassays and biosensing technologies.

## Highlights

• Fc glycosylation influences IgG susceptibility to chemical reduction hinge region.
• IgG deglycosylation enhances hinge region chemical reduction yields.
• IgG deglycosylation overcomes IgG batch variability in chemical reduction.
• IgG deglycosylation standardizes chemical reduction in bioconjugation protocols.

## Graphical abstract

**Figure:**
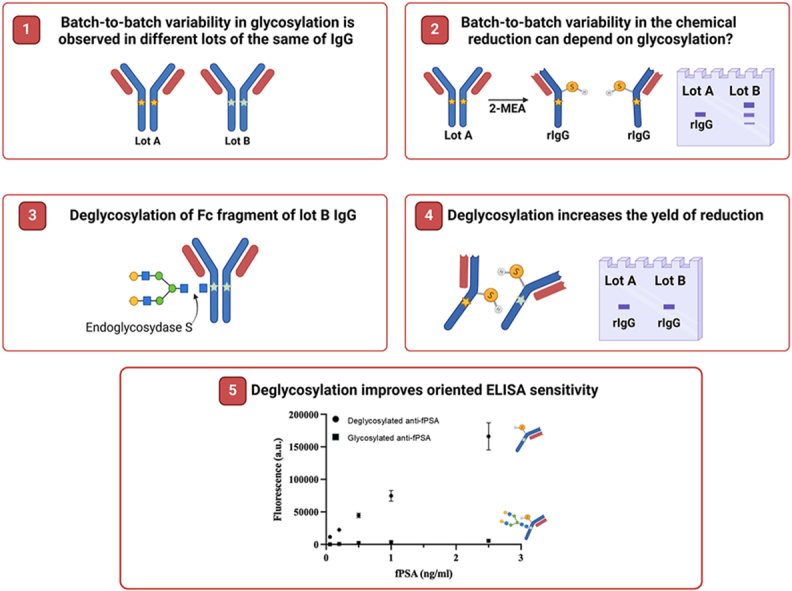

## 1 Introduction

Antibodies are globular glycoproteins of the immune system that play a central role in activating both innate and adaptive immune responses upon binding to specific antigens. Their functionality arises from a unique structure comprising the Fab and Fc fragments, connected by a hinge region. The Fab fragment mediates antigen binding, while the Fc fragment interacts with Fcγ receptors (FcγRs) to trigger various cellular effector functions [1,2]. This structural organization is particularly advantageous in immunoassays, where the Fab detects target analytes of interest and the Fc fragment enables antibodies immobilization on the surface of microplates.

**Fig. 1:**
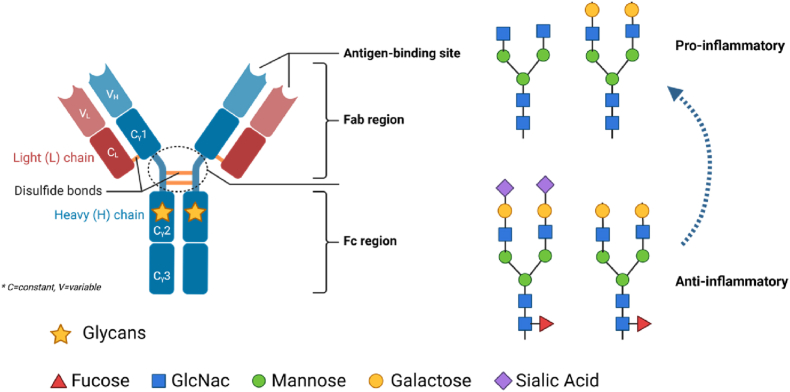
Immunoglobulin G structure and possible glycosylation structures of the C_H_2 domain.

Among the various classes of antibodies, immunoglobulins G (IgGs, 150 kDa) is preferred in immunoassays due to their higher specificity and affinity for antigens compared to the other classes of antibodies. The Fc structure of IgGs consists of two identical heavy chains, with two constant immunoglobulin domains of which the C_H_2 domain contains a single glycosylation site. The glycan sequence in the C_H_2 domain comprises a heptasaccharide core made up of four N-acetylglucosamine and three mannose residues. The core can be further modified by the addition of fucose to the N-acetylglucosamine residues, and/or by the addition of galactose and/or sialic acid at the branches [3,4] (Fig. 1).

In IgGs, glycans serve a structural role by stabilizing the C_H_2 domain thereby defining the overall structure of the Fc fragment. Small-angle X-ray scattering experiments conducted in solution, a physiologically relevant environment, demonstrated that deglycosylated Fc fragments exhibit larger radii of gyration compared to their glycosylated counterparts, indicating a more open C_H_2 conformation [5]. This observation was corroborated by X-ray crystallography of the murine unglycosylated Fc fragment, where Feige et al. reported increased mobility and significant displacement of the C′E loop, the glycan attachment site, relative to fully glycosylated structures [6]. Similarly, size exclusion chromatography revealed that deglycosylated Fc fragments display an increased hydrodynamic radius, attributed to a greater separation between the two C_H_2 domains of the Fc fragment compared to the glycosylated form [7]. Moreover, NMR spectroscopy [8] detected significant chemical shift changes in the C′E loop region upon Fc deglycosylation, confirming that glycans stabilizes local C_H_2 conformations and limit loop flexibility.

Additionally, deglycosylation has been associated with enhanced susceptibility of the hinge region to enzymatic cleavage, as reported by Raju et al. [9]. Together, these structural insights demonstrate that removal of Fc glycans induces C_H_2 domain rearrangements that may affect hinge region accessibility. In physiological conditions, the glycan composition of the Fc fragment modulates IgG affinity for Fcγ receptors (FcγRs), influencing the intensity of effector functions. It was observed that deglycosylation reduces IgG affinity for FcγRs, impairing the ability of antibodies to induce cellular effector functions. Indeed, deglycosylated IgGs lack immune effector functions such as antibody-dependent cellular cytotoxicity (ADCC) [8,[10], [11], [12], [13]]. The hinge region of antibodies is in proximity of the C_H_2 domain, and it is a flexible amino acid stretch, which links, through disulfide bonds, the two Fab regions of IgGs. Bioconjugation chemistry and surface functionalization in immunoassays often require the selective reduction of disulfide bonds within the hinge region using a mild reducing agent, such as 2-mercaptoethylamine (2-MEA). This approach generates half antibody containing a monovalent antigen Fab region extended with Fc C_H_2 and C_H_3 domains (rIgG, 75 kDa). rIgG exposes a thiol group that can be conjugated with enzymes, nano-particles, or modified surfaces via sulfhydryl residues exposed in the hinge region. This strategy represents one of the site-specific coating methods that orients IgGs with the Fab region facing outward, thereby enhancing antigen-binding capacity and improving the sensitivity of enzyme-linked immuno-sorbent assays (ELISA)[[14], [15], [16], [17]].

The structural changes induced by deglycosylation can be particularly relevant for bioconjugation chemistry and surface functionalization based on rIgG approach. Indeed, it is reasonable to infer that modifications in the C_H_2 domain of deglycosylated IgGs can affect the efficiency of selective chemical reduction in the hinge region. This study aimed to evaluate the reduction efficiency of disulfide bonds in both glycosylated and deglycosylated IgGs. While research on IgG glycosylation has primarily focused on its physiological role in vivo and its implications in biotechnology, particularly in the development of therapeutic antibodies [[18], [19], [20]]. However, our findings demonstrate that Fc deglycosylation significantly enhances the efficiency of chemical reduction in the hinge region, overcoming batch-to-batch variability in glycan composition across different IgG lots. These results extend the applicability of the rIgG approach in bioconjugation and immunoassay development. The increased susceptibility to 2-MEA observed in our study is likely due to conformational changes in the C_H_2 domain following deglycosylation, which enhance the accessibility and structure of the hinge region.

## 2 Materials and methods

### 2.1 Chemicals

Mouse monoclonal antibody directed towards the free prostate specific antigen (anti-fPSA) lot A and lot B, and VIDAS® fPSA assay ref. 30440 were supplied by bioMérieux Italia (Bagno a Ripoli, Italia). Pierce™ Maleimide Activated Plates were purchased by Thermo Fisher Scientific (Waltham, MA, USA). Sephadex G-25 in PD-10 desalting columns (GE Healthcare, Little Chalfont, UK), 2-Mercaptoethylamine (2-MEA), ethylenediaminetetraacetic acid disodium salt (EDTA), Coomassie Brillant Blue R-250, Ca2+ and Mg2+ free Dulbecco's Phosphate Buffered Saline (PBS), fat free milk and Tween-20 were purchased from Sigma Chemical Co. (St. Louis, MO, USA). Endo S and chitin magnetic beads were purchased by New England Biolabs (Ipswich, MA, USA). Tris(hydroxymethyl)aminomethane (TRIS), TRIS-HCl, sodium dodecyl sulphate (SDS), acrylamide-bisacrylamide 30 %, Tetramethylethylenediamine (TEMED) and Ammonium persulfate (APS) were acquired by Bio-Rad Laboratories, Inc. (Hercules, CA). Deionized water purified by a Millipore Milli-Q gradient system (>18 MΩ cm) was used in all the experiments.

### 2.2 Antibody deglycosylation

Anti-fPSA IgGs were deglycosylated using Endo S, an endoglycosidase known for its high specificity towards N-linked glycans in native IgGs. 100 μg anti-fPSA antibodies were incubated with 1 μl of 10X GlycoBuffer and 1 μl of Endo S conjugated with chitin binding domain (CBD) for 60 min at 37 °C, according to the instruction of the manufacturer. Deglycosylated antibodies were purified by incubating the solution with 50 μl of chitin magnetic beads for 10 min at 4 °C under mild agitation. Magnetic chitin beads were washed 3 times with 50 μl of 50 mM NH4 formate buffer pH 4.4 collecting the supernatant. Deglycosylation was verified by 8 % SDS-PAGE and protein bands were visualized with Coomassie Brillant Blue R-250.

### 2.3 Antibody reduction by 2-MEA

Antibody were reduced according to the protocol described by Hermanson [21].

1 mg/ml anti-fPSA were reduced by 53 mM 2-MEA in PBS with 10 mM EDTA. The reduction mixture was incubated at 37 °C for 90 min under mild agitation. Then, the reducing agent and EDTA were removed using a Sephadex G-25 in PD-10 desalting column following the instruction of the manufacturer. The presence of reduced anti-fPSA (75 kDa) was assessed by non-reducing 8 % sodium dodecyl sulphate poly-acrylamide gel electrophoresis (SDS-PAGE); protein bands were stained with Coomassie brilliant blue R-250.

### 2.4 ELISAs performed with glycosylated or deglycosylated rIgG on maleimide activated microplates

To verify if the improved efficiency of the chemical reduction of deglycosylated anti-fPSA enhances the performances of ELISA based on oriented IgGs, glycosylated or deglycosylated reduced anti-fPSA were tested in ELISA performed on maleimide activated microplate.

Maleimide activated microplates were coated with 10 μg/ml glycosylated or deglycosylated reduced anti-fPSA in PBS-EDTA (100 μl). After O/N incubation at 4 °C, coating solution was removed by three washing steps with PBS containing 0.01 % (w/v) TWEEN® 20 (PBS-T) (200 μl). Nonspecific binding of maleimide-activated microplates was blocked by 1 h incubation at room temperature with freshly prepared 10 μg/ml cysteine in PBS (200 μl). Samples, consisting of dilutions of purified fPSA standard (0.06 ng/ml, 0.2 ng/ml, 0.5 ng/ml, 1 ng/ml, and 2.5 ng/ml), were incubated for 1 h at room temperature. Unbound antigen was then removed by three washing steps. PBS was used as a negative control. 100 μl of detection antibodies conjugated with alkaline phosphatase (ALP), available in the VIDAS® kits, were incubated for 1 h at room temperature. After three washing steps, 200 μl/well of ALP fluorescent substrate (VIDAS® Optical Substrate) was added. The generated fluorescence was measured after 20 min incubation time on BioTek Synergy H1 plate reader (Agilent Technologies, Santa Clara, CA, USA) setting excitation wavelength at 360 nm and emission at 450 nm. All incubation steps were carried out at room temperature.

### 2.5 VIDAS® fPSA assay performed with glycosylated or deglycosylated rIgG

To assess the impact of glycan composition on the chemical reduction efficiency across different antibody lots, we chemically reduced anti-fPSA lot number 2, and the resulting rIgGs were then applied in the VIDAS® fPSA assay as described below.

Six replicates of both commercial VIDAS® fPSA assay and oriented VIDAS® fPSA assay based on rIgGs were performed. Calibrator S1 of VIDAS® fPSA assay diluted 1:10 in PBS (0.8 ng/ml) was used as sample.

## 3 Results & discussion

### 3.1 Glycosylation and IgG reduction efficiency

**Fig. 2:**
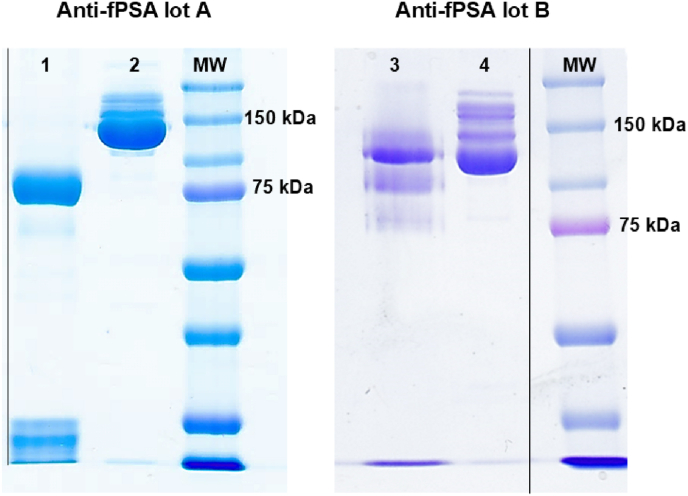
SDS-PAGE under non reducing condition of two different lots of IgG anti-fPSA. Lane 1: reduced anti-fPSA IgG lot A; Lane 2: non reduced anti-fPSA IgG lot A; Lane 3: reduced anti-fPSA IgG lot B; Lane 4: non reduced anti-fPSA IgG lot B. MW: molecular weight; black lines: image crop, see supporting information.

Two different lots of the same monoclonal mouse antibody targeting free prostate specific antigen (anti-fPSA) were chemically reduced using 2-MEA to produce rIgGs. Under non-reducing conditions, SDS-PAGE analysis typically shows monovalent rIgGs as a band at approximately 75 kDa, corresponding to the complex of a heavy chain with its light chain. As shown in Fig. 2, lot A (lane 1) presented a distinct band at the expected molecular weight of 75 kDa indicating successful and complete reduction. In contrast, lot B (lane 3) displayed multiple bands ranging from 75 to 100 kDa suggesting incomplete reduction. Despite its straightforward execution, chemical reduction using 2-MEA is not widely adopted in immunoassay development. One reason may be the variability in rIgG yield observed between different lots of the same IgG clone. This variability likely arises from differences in the glycan composition of IgGs, which are influenced by the culture conditions of the hybridoma secreting the antibodies [22]. In fact, glycan composition is known to affect the C_H_2 domain structure altering its conformation and folding [5,6]. It is plausible that, in lot B, specific glycan compositions induce a C_H_2 domain folding that reduces the exposure of disulfide bonds in the neighboring hinge region to reducing agents, thereby limiting the susceptibility of IgGs to chemical reduction. In this context, glycosylation may restrict the implementation of IgG orientation strategies that depend on hinge region sulfhydryl groups for immobilization in immunoassays.

Alternatively, the lower reduction yield observed in lot B could be due to IgG aggregates or high-molecular-weight complexes, potentially influenced by differences in storage buffer composition between lots. However, the SDS-PAGE depicted in Fig. 2 did not reveal any significant differences in IgG aggregation or high-molecular-weight species between lots A and B.

### 3.2 Impact of deglycosylation on IgG reduction efficiency

**Fig. 3:**
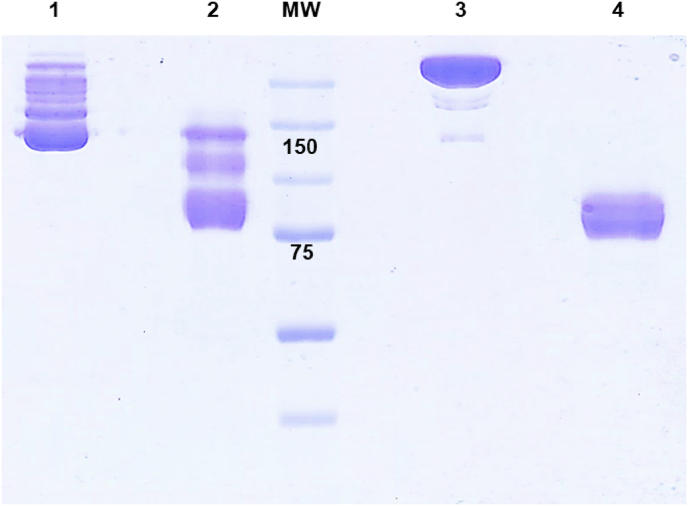
8 % SDS-PAGE under non reducing condition of glycosylated and deglycosylated anti-fPSA. Lane 1: glycosylated non reduced anti-fPSA; Lane 2: glycosylated reduced anti-fPSA; Lane 3: deglycosylated non reduced anti-fPSA; Lane 4: deglycosylated reduced anti-fPSA.

To evaluate whether the specific glycan composition of anti-fPSA lot B affects the chemical reduction of the hinge region, N-linked glycan side chains in the C_H_2 domain were enzymatically removed using Endo S, as described in section 3.2. Both glycosylated and deglycosylated anti-fPSA samples were subsequently reduced with 2-MEA, and their reduction patterns were analyzed by SDS-PAGE under non-reducing conditions. As shown in Fig. 3 (lanes 1 and 2), the glycosylated anti-fPSA displayed multiple bands between 150 and 75 kDa, indicating incomplete reduction. In contrast, the deglycosylated anti-fPSA exhibited the expected single band at 75 kDa, as observed for anti-fPSA lot A. These findings demonstrate that deglycosylation enhances the susceptibility of the hinge region to chemical reduction in antibodies with glycan compositions that restrict access to the reducing agent. This observation aligns with previous reports [9], which suggest that glycan removal from the C_H_2 domain promote a Fc open conformation, improving accessibility to the hinge region for both enzymatic cleavage and chemical reduction. Moreover, implementing a deglycosylation step may address lot-to-lot variability in rIgG production, offering a strategy to standardize yields across different antibody batches.

### 3.3 Effect of deglycosylation on immunoassay sensitivity

**Fig. 4:**
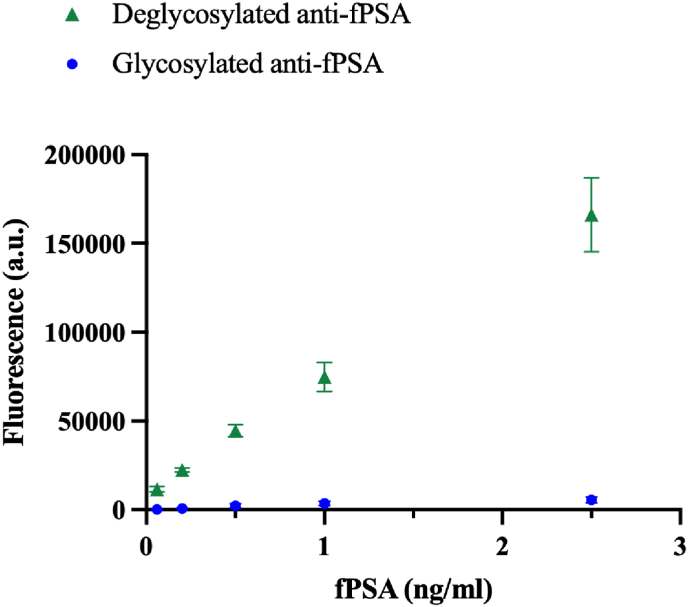
ELISA for the detection of fPSA based on oriented IgGs performed with deglycosylated IgGs (triangle, ) and glycosylated IgGs (circle, ).

To evaluate the effect of improved rIgG yield of anti-fPSA lot B on ELISA sensitivity, both glycosylated and deglycosylated anti-fPSA antibodies (lot B) were reduced and tested in an in-house ELISA for fPSA detection, as outlined in section 3.4. Five concentrations of fPSA, ranging from 0.06 ng/ml to 2.5 ng/ml, were analyzed. The specificity of the assay, as reported by the manufacturer, is 78.21 % (95 % confidence interval: 67.61–86.05 %). The absence of nonspecific binding was verified in our previous study [23]. As shown in Fig. 4, the deglycosylated anti-fPSA antibodies significantly enhanced ELISA sensitivity, yielding at least a 20-fold increase in the fluorescent signal across all tested concentrations. The results suggest that IgG deglycosylation improves rIgG production, leading to a higher proportion of IgGs that can be efficiently oriented on surfaces. This increased availability of rIgGs enhances antigen binding efficiency, resulting in improved assay sensitivity.

### 3.4 Application of deglycosylation step in commercial immunoassays

It has been shown that the sensitivity of immunoassays can be significantly enhanced by orienting IgGs via direct binding of rIgGs to gold-functionalized surfaces. This approach was successfully applied in the commercial VIDAS® fPSA assay, as described in our previous work [23] where reduced anti-fPSA antibodies (lot A) were oriented by direct binding to gold surfaces. To evaluate whether deglycosylation could be effectively applied to the development of commercial immunoassays, glycosylated and deglycosylated anti-fPSA lot B were chemically reduced with 2-MEA. The resulting rIgGs were applied in the VIDAS® fPSA assay and compared with the commercial VIDAS® fPSA assay using 0.8 ng/ml of fPSA as a sample, as described in section 3.5.

As shown in Table 1, the modified VIDAS® fPSA assay using glycosylated rIgG from lot B failed to enhance the fluorescent signal compared to the commercial assay, showing a lower signal. This likely depends on the presence of partially reduced IgGs, which decrease the antigen binding capability of the assay surface. Conversely, the modified assay using deglycosylated rIgGs demonstrated a significantly higher fluorescent signal compared to the commercial assay. These findings confirm that deglycosylation improves the accessibility of the hinge region for chemical reduction, enhancing rIgG production. Consequently, this leads to a greater proportion of rIgGs being properly oriented on the surface, restoring the enhanced sensitivity previously observed using anti-fPSA lot A [23].

**Table 1:** Fluorescent signal (mean ± SD, n = 6) obtained using glycosylated or deglycosylated rIgG anti-fPSA lot B, compared to the commercial assay.

Assay type	Fluorescent signal (a.u.)
Commercial VIDAS® fPSA	329 ± 2.89
Modified VIDAS® fPSA - glycosylated rIgG	166 ± 19.34
Modified VIDAS® fPSA - deglycosylated rIgG	590 ± 13.98

Overall, results highlight that deglycosylation mitigates batch-to-batch variability in the chemical reduction of IgGs, which arises from differences in glycan composition that affect hinge region accessibility. Implementing a deglycosylation step prior to chemical reduction significantly reduces variability, resulting in more consistent rIgG yields. This improvement enhances reliability and broadens the applicability of orientation techniques involving chemical reduction, making them more suitable for immunoassay development. In therapeutic applications it is described that the stability of deglycosylated antibodies can be influenced by the removal of glycans, which may affect the conformational stability of the C_H_2 domain. Studies have shown that deglycosylated antibodies exhibit less thermal stability for the C_H_2 domain and reduced resistance to chemical-induced unfolding [7]. However, the overall stability of coated deglycosylated antibodies in immunoassays is comparable to their glycosylated counterparts, largely depending on the stabilizing and blocking agents employed during assay preparation. Our deglycosylation approach utilizes commercially available reagents, such as Endo S conjugated with a chitin-binding domain and chitin-conjugated magnetic microparticles, facilitating straightforward and rapid antibody deglycosylation and purification. This streamlined process supports scalability, enabling application across various assay formats and production scales. Furthermore, employing standardized, commercially available products for deglycosylation enhances protocol repeatability.

However, this study has some limitations. The assessment of anti-fPSA deglycosylation and the relative yield of deglycosylated versus glycosylated IgGs was qualitative. Future studies should aim to quantitatively analyze rIgG production from both forms. Additionally, this investigation focused on a single IgG clone. Expanding the analysis to different IgG subclasses would provide valuable insights, particularly as hinge region disulfide bonds differ across subclasses, potentially affecting their susceptibility to chemical reduction.

## 4 Conclusions

While there is significant research on the role of glycosylation in antibody function and therapeutic antibodies, studies specifically focused on the direct application of deglycosylated antibodies in immunoassay development remain less common. This study explored how IgG deglycosylation affects susceptibility to chemical reduction, facilitating the generation of rIgGs for use in orientation approaches in immunoassay development. The findings suggest that deglycosylation enhances rIgG production, leading to a higher proportion of antibodies that can be efficiently oriented on surfaces. This, in turn, improves antigen binding efficiency and enhances assay sensitivity. Moreover, deglycosylation effectively addresses batch-to-batch variability in the chemical reduction of different antibody lots of the same IgG clone. This variability arises from differences in glycan composition, which affect the structure and conformation of C_H_2 domain, thereby influencing the accessibility of the nearby hinge region to chemical reduction. To date, in vitro diagnostic companies have not widely adopted this technique due to reproducibility concerns across different lots of the same IgG clone. Our findings suggest that deglycosylation is a promising strategy to standardize the production of rIgGs, mitigating the batch-to-batch variability in chemical reduction efficiency. This approach has the potential to improve the reproducibility of immunoassays across different lots, paving the way for more reliable and sensitive diagnostic tools. Additionally, Fc deglycosylation could impact the efficiency of other bioconjugation techniques involving antibodies, such as their conjugation to enzymes, nanoparticles, and fluorophores.

## CRediT authorship contribution statement

**Vanessa Susini:** Writing – original draft, Conceptualization. **Silvia Ursino:** Methodology. **Chiara Sanguinetti:** Investigation. **Alice Botti:** Investigation. **Laura Caponi:** Resources. **Maria Franzini:** Writing – review & editing, Supervision.

## Declaration of generative AI and AI-assisted technologies in the writing process

During the preparation of this work the authors used ChatGPT to improve the readability and language of the manuscript. After using this tool, the authors reviewed and edited the content as needed and took full responsibility for the content of the published article.

## Funding

This work was supported by project “Hub multidisciplinare e interregionale di ricerca e sperimentazione clinica per il contrasto alle pandemie e all'antibioticoresistenza (PAN-HUB)” funded by the 10.13039/501100003196Italian Ministry of Health (POS 2014–2020, project ID: T4-AN-07, CUP: I53C22001300001) and by institutional University funds.

## Declaration of competing interest

The authors declare that they have no known competing financial interests or personal relationships that could have appeared to influence the work reported in this paper.

## Data Availability

All data are contained within the article and in the supporting information.
