# Supplementary material for: The impact of Fc glycosylation on IgG susceptibility to hinge region chemical reduction: implications for the development of immunoassays
Source: Biochem Biophys Rep. 2025 Jun 25;43:102112. doi: 10.1016/j.bbrep.2025.102112 (PMC12246625; doi:10.1016/j.bbrep.2025.102112)
Supplement: Multimedia component 1 [file mmc1.docx]

# The impact of Fc glycosylation on IgG susceptibility to hinge region chemical reduction: implications for the development of immunoassays.

Vanessa Susini*, Silvia Ursino, Chiara Sanguinetti, Alice Botti, Laura Caponi, Maria Franzini.

Department of Translational Research and of New Surgical and Medical Technologies, University of Pisa, via Savi 10, Pisa, Italy

*Corresponding author:

Vanessa Susini

Dep. of Translational Research and of New Surgical and Medical Technologies, University of Pisa, Pisa,

Via Savi 10, 56126 Pisa

e-mail address: [vanessa.susini@unipi.it](mailto:vanessa.susini@unipi.it)

**0
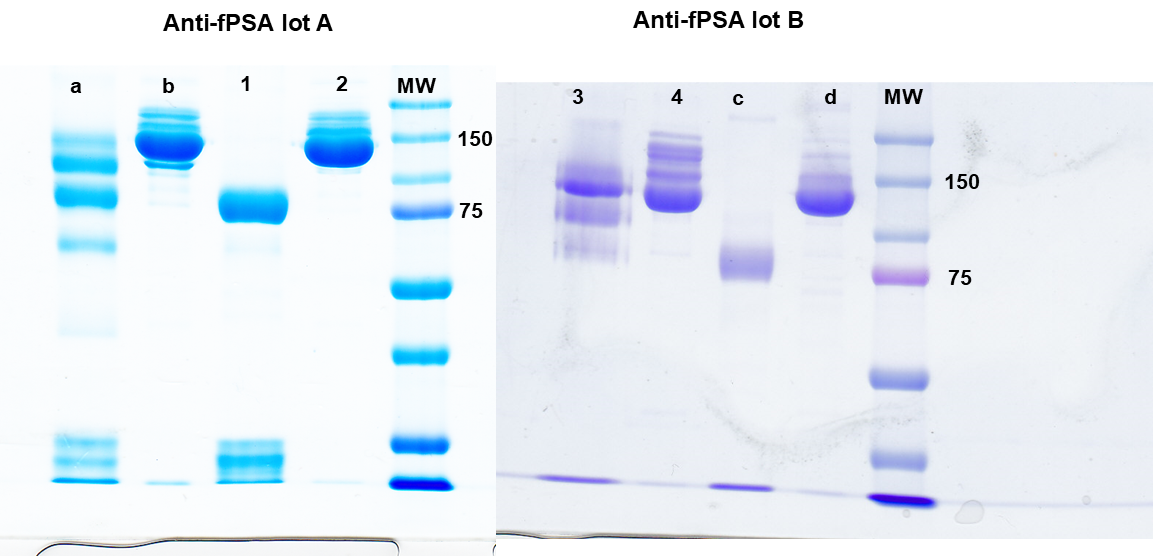
**

**Figure 1 – original.** SDS-PAGE under non reducing condition of two different lots of IgG anti-fPSA.

In the manuscript are shown Lane 1: reduced anti-fPSA IgG lot A; Lane 2: non reduced anti-fPSA IgG lot A; Lane 3: reduced anti-fPSA IgG lot B; Lane 4: non reduced anti-fPSA IgG lot B. MW: molecular weight.

The lanes a and b cropped in the manuscript represented respectively the reduced anti-α-fetoprotein antibody and the corresponding non reduced antibody.

Lanes c and d cropped in the manuscript represented respectively the reduced anti-cardiac troponin I antibody and the non reduced ones.

Anti-α-fetoprotein and anti-cardiac troponin I antibody were tested for their susceptibility to the chemical reduction by 2-MEA as described in the Material and Metod paragraph of the manuscript, but we chose to focus on the two different batches of the same monoclonal anti-fPSA antibody.


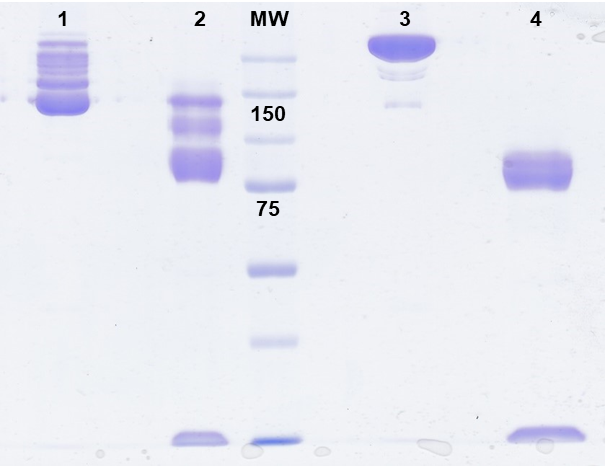


**Figure 2** – **original**. 8% SDS-PAGE under non reducing condition of glycosylated and deglycosylated anti-fPSA. Lane 1: glycosylated non reduced anti-fPSA; Lane 2: glycosylated reduced anti-fPSA; Lane 3: deglycosylated non reduced anti-fPSA; Lane 4: deglycosylated reduced anti-fPSA.
